# 

**Published:** 1888-03-10

**Authors:** 


					rrmpE C7TTE?PtTmTT
A ??A
r Vol hi-
March 10th, 1888.
e8l*tered at the Creneral Post Office
as a Newspaper.]
.
0
Per Annum, 6s. ed.. post free.
Monthly Farts, 6d.; post free, 7id.
Ditto per Ann., 7s. ad. post free.
A Weekly Institutional Journal of
Science, flfceMcine, anfc flWlantbropE.

				

